# Efficacy and Safety of Antibiotic Impregnated Microporous Nanohydroxyapatite Beads for Chronic Osteomyelitis Treatment: A Multicenter, Open-Label, Prospective Cohort Study

**DOI:** 10.3390/antibiotics12061049

**Published:** 2023-06-15

**Authors:** Chittawee Jiamton, Adinun Apivatgaroon, Saree Aunaramwat, Banchai Chawalitrujiwong, Chaiwat Chuaychoosakoon, Sitthiphong Suwannaphisit, Choen Jirawison, Chonlathan Iamsumang, Pinkawas Kongmalai, Pawaris Sukvanich, Pongtep Na Nakorn, Worawit Ongbumrungphan, Pawin Rattanasumrit, Suthee Tharakulphan, Thanachai Thongtanworapat, Faungchat Thammarakcharoen, Autcharaporn Srion, Jintamai Suwanprateeb, Bancha Chernchujit

**Affiliations:** 1 Institute of Orthopaedics, Lerdsin Hospital, Silom Road, Bang Rak, Bangkok 10500, Thailand; cjiamton@gmail.com; 2 Queen Savang Vadhana Memorial Hospital, Jerm Jom Phon Road, Tambon Si Racha, Si Racha, Chonburi 20110, Thailand; 3 Department of Orthopaedics, Faculty of Medicine, Thammasat University, Paholyothin Road, Klong Nueng, Klong Luang, Pathum Thani 12121, Thailand; adino_ball@yahoo.com; 4 Paholpolpayuhasena Hospital, Sangchuto Road, Pak Phraek, Mueang, Kanchanaburi 71000, Thailand; tenghai111@yahoo.com; 5 Suppasitthiprasong Hospital, Sappasit Road, Nai Mueang, Mueang, Ubon Ratchathani 34000, Thailand; banchaicha2511@gmail.com; 6 Department of Orthopedics, Faculty of Medicine, Prince of Songkla University, Karnjanavanich Road, Kho Hong, Hat Yai, Songkhla 90110, Thailand; psu.chaiwat@gmail.com (C.C.); susitthi@medicine.psu.ac.th (S.S.); 7 Bhudasothon Hospital, Marupong Road, Na Mueang, Mueang, Chachoengsao 24000, Thailand; choencj@gmail.com; 8 Lampang Hospital, Phahonyothin Road, Hua Wiang, Mueang, Lampang 52000, Thailand; bonecorrector@hotmail.com; 9 Department of Orthopedics, Faculty of Medicine, Srinakharinwirot University, Rangsit-Nakhon Nayok Road, Baan na, Ongkharak, Nakhon Nayok 26120, Thailand; pinkawass@hotmail.com (P.K.); paosukvanich@gmail.com (P.S.); 10 Hatyai Hospital, Ratthakan, Tambon Hat Yai, Hat Yai, Songkhla 90110, Thailand; phongtap@hotmail.com; 11 Bhumibol Adulyadej Hospital, Phahonyothin Road, Sai Mai, Bangkok 10220, Thailand; worawit_md@hotmail.com (W.O.); farmpawin@gmail.com (P.R.); 12 Khon Kaen Hospital, Sri Chant Road, Nai Mueang, Mueang, Khon Kaen 40000, Thailand; oe_orthokkh@hotmail.com; 13 Pathumtani Hospital, Lat Lum Kaeo Road, Bang Prok, Muang, Pathum Thani 12000, Thailand; thanachai.ong@hotmail.com; 14 National Metal and Materials Technology Center (MTEC), National Science and Technology Development Agency (NSTDA), 111 Phahonyothin Road, Khlong Nueng, Khlong Luang, Pathum Thani 12120, Thailand; faungcht@mtec.or.th (F.T.); autcharas@mtec.or.th (A.S.); 15 Thammasat University Center of Excellence in Computational Mechanics and Medical Engineering, Thammasat University, Phahonyothin Road, Khlong Nueng, Khlong Luang, Pathum Thani 12121, Thailand

**Keywords:** osteomyelitis, infection, localized antibiotics delivered beads, hydroxyapatite

## Abstract

Chronic osteomyelitis is still a serious health problem that causes disabling conditions and has an impact on the quality of life. The objective of this study was to determine the clinical efficacy and safety of localized antibiotics delivery via impregnated microporous nanohydroxyapatite (nHA-ATB) beads for chronic osteomyelitis treatment. A total of 62 patients were enrolled in this study. After radical surgical debridement, the bone defect was filled with three types of antibiotics (vancomycin or gentamicin or fosfomycin) impregnated HA beads. The follow-up period was 48 weeks. It was found that the success rate was approximately 98% with a re-infection in only one patient. Quality of life of all patients after treatment improved significantly over time. Systemic exposure to vancomycin and gentamicin after beads implantation was limited and high local antibiotics concentrations were found in wound drainage fluid at 24, 48 and 72 h. Blood biochemistry measurements did not show any nephrotoxic or hepatotoxic effects. 20 adverse events were reported, but 90% of the events were resolved without having to remove the beads and the patients recovered. Satisfactory outcomes were observed in terms of success rate, quality of life and adverse effect. nHA-ATB beads impregnated by vancomycin or gentamicin or fosfomycin could potentially be employed as an alternative product of choice for localized antibiotics delivery in chronic osteomyelitis treatment.

## 1. Introduction

Chronic osteomyelitis is one of the common orthopedic problems that can cause disabling conditions and have an impact on the quality of life globally. The cost of infected bone treatment was estimated to be 1.2-fold to six-fold higher than those of non-infected cases [1]. The exact epidemiology of osteomyelitis is largely unknown and varied by several factors including counties, ages, locations, and risk factors. The incidence of pediatric osteomyelitis in high-income countries is 1.94–13 per 100,000 populations is lower than that of low-income countries which is 43–200 per 100,000 populations [2]. The annual prevalence and incidence of pediatric osteomyelitis were reported to be 20 and 9.2–13 cases per 100,000 individuals respectively [3,4,5,6]. The prevalence of osteomyelitis in Germany was 16.7 cases per 100,000 population in 2018 which increased by 10.44% from 2008 [7] while the incidence of osteomyelitis in the United States was reported to be 90 cases per 100,000 patients per year [8]. Osteomyelitis in diabetic patients could be as high as 10–20% [9,10].

Several factors, including hematogenous spread and direct contamination through an open wound, particularly open fractures, might contribute to this complication [11,12,13]. The patients usually present with recurrent sinus tract, pain, and delayed healing of the fracture. The general principles of chronic osteomyelitis treatment include adequate surgical debridement to remove all non-vitalized tissue and dead bone, adequate soft tissue coverage and administration of systemic antibiotics. Nevertheless, the limited blood circulation of bone tissue and the secretion of biofilm in the infected area results in poor antibiotic penetration at the infected bone resulting in the remnant of microorganisms in the necrotic bone tissue, especially in the area that has not undergone adequate surgical debridement. High dose and prolonged systemic antibiotics are thus often administrated for effective eradication of the bacteria which may induce systemic toxicity to the patients. To overcome these risks, local antibiotics delivery in the form of antibiotics loaded beads have been shown to be an effective solution for providing a sustained, local, high concentration of antibiotics at the site of infection and lower systemic side effects compared with intravenous antibiotics [14,15,16,17]. Although no clear agreement on the advantage of local antibiotic beads over intravenous antibiotics has been established, this localized treatment plays a vital role in treatment strategy especially in chronic osteomyelitis [18].

Polymethylmethacrylate (PMMA) has been long used as a local antibiotic released carrier and is commercially available as a ready-to-use antibiotic containing bead chain or antibiotic bone cement for manual bead preparation. It is a nonbiodegradable material that needs a second operation for bead removal and generates heat during the reaction which limits the use of only heat-stable antibiotics [19]. There was also a concern about the increase of bacterial antibiotic resistance of antibiotic-loaded PMMA, and a local substratum for bacterial inoculation on its surface although there is no strong clinical evidence for these concerns [20,21,22]. Moreover, the use of PMMA could induce foreign body reactions and there might be a need for bone graft to fill the defect. To overcome these disadvantages, various materials including calcium sulfate [23,24,25,26], polycaprolactone [27,28], polylactide/polyglycolide [29,30], chitosan [31], calcium sulfate/calcium carbonate [32,33] were extensively investigated as alternatives. Among these, pure calcium sulfate and a mixture with calcium carbonate or hydroxyapatite were commercially available and have been increasingly used in place of PMMA. However, there is still not a prominent level of clinical evidence and studies that can explicitly prove which materials are superior to other materials.

Concerning synthetic bone graft substitutes for bone defect reconstruction, calcium phosphates, particularly hydroxyapatite (HA), is probably the most popular due to its resembled chemical composition to the inorganic constituent of bone. It is a highly biocompatible material and possesses osteoconductive properties. It is typically used as a bone substitute to regenerate a new bone in the defect area. In recent years, HA has been investigated as a local antibiotic delivery system since it could provide a bone regeneration capability by providing the osteoconductive structure that helped filling dead space and promoted new tissue ingrowth which in turn led to the repair of osseous defects while releasing high concentration of antibiotics. This eliminated the need for subsequent removal of the antibiotics carrier and the use of additional grafting materials [34,35,36,37]. However, it was also noted that the antibiotic elution profile or biodegradation profile was not efficient and predictable for local drug release which might be partly due to the ability of antibiotics to bind on its dense surface only [38,39,40]. Typical HA was generally fabricated by high temperature sintering technique resulting in pore-less microstructure with high crystallinity and low resorption rate. Recently, low temperature phosphorization of calcium sulfate to HA was developed as a new technique to produce low crystalline nanostructure hydroxyapatite (nHA) which is closer to that of natural bone [41,42]. In comparison to the typical high-temperature sintering route, this low-temperature route produced microporous nano-hydroxyapatite with osteoconductivity and resorbability due to its nanostructure and low crystallinity. This material has been evaluated for its purity of composition, toxicity, biocompatibility with bone cells, microstructure, in vivo safety and clinical efficacy as bone graft and also its capability for antibiotics impregnation for using as locally delivered antibiotic bead [43,44]. Due to its numerous interconnected micropores, antibiotics could be absorbed throughout the matrix, retained and released more effectively compared to the surface-bound antibiotics as in the case of typical sintered hydroxyapatite. In medical device development, the clinical evaluation is an essential step to demonstrate the safety and performance of the medical device for either pre-marketing or post-marketing purposes. Due to their market availability, a vast number of literature reviewing or reporting the clinical evaluation of commercial PMMA and calcium sulfate for local antibiotics delivery was noted [19,23,24,25,26,32,33], but the clinical evaluation of using hydroxyapatite as a local antibiotic carrier was still scarce and performed in a small number of patients [45,46,47,48,49]. This work is aimed to expand the knowledge of using calcium phosphate as a local antibiotic carrier and; in particular, to determine the efficacy and safety of using developed nHA-ATB for chronic osteomyelitis treatment in a large group of patients and in a multicenter setting in terms of success rate, wound status, antibiotic side effect, quality of life of the patient and adverse event. The knowledge gained from this study can be useful for justifying the use of nHA-ATB, revealing its advantages and drawbacks, and elucidating the precautions that should be taken when this material is used in the clinical setting for osteomyelitis treatment.

## 2. Results

### 2.1. Demographics and Clinical Characteristics

**Figure 1:**
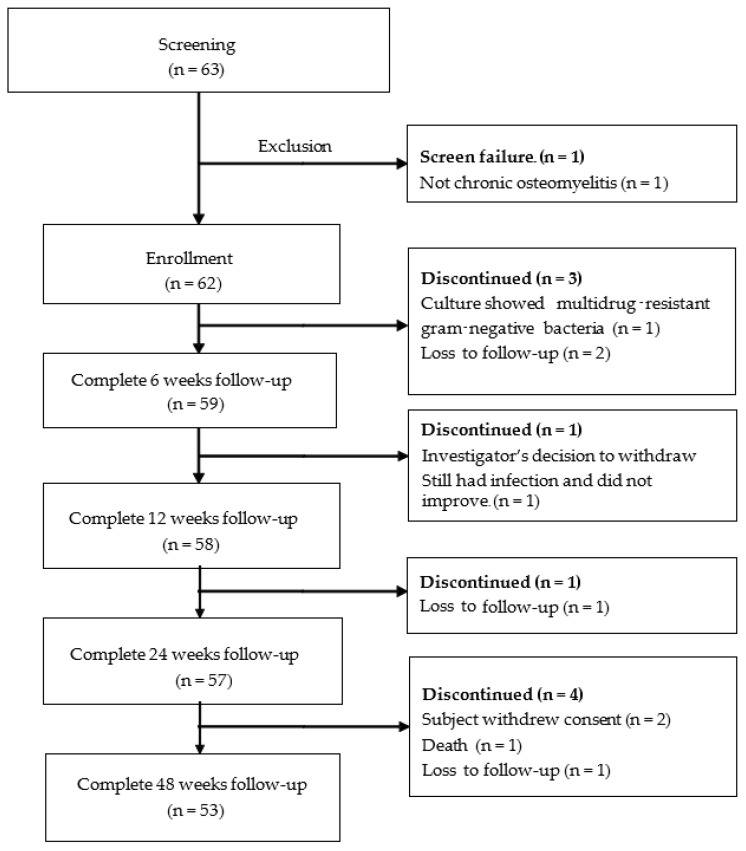
CONSORT flowchart of the study.

Sixty-three patients were eligible for the study. One patient was excluded as the condition was not fulfilled to chronic osteomyelitis. Sixty-two patients were thus enrolled for treatment. Nine patients were discontinued from the study due to (1) Infected with multidrug-resistant gram-negative bacteria from an intra-operative culture (one patient), (2) discontinued by the decision of the investigator due to the presence of acute on-top chronic osteomyelitis and the clinical outcome was not improved (one patient), (3) withdrew their consent due to inconvenience in traveling (two patients), (4) loss to follow-up (four patients), and (5) died from other causes (one patient). Therefore, 53 patients participated in the whole 48 weeks follow-up in the study. Diagram of the study flow according to CONSORT guideline is shown in Figure 1. The demographic data including sex, age, wound location, Cierny-Mader (C-M) classification (anatomic type, physiological condition and risk factors), causes, and history of previous surgery for osteomyelitis are summarized in Table 1. Forty-nine patients (79%) were male. The mean age was 47.2 years. The major cause of osteomyelitis was post-traumatic injury in 48 patients (77.4%) while 14 patients (22.6%) were infected from hematogenous spreading. Fifteen patients (24.2%) never had a history of previous surgery for this condition while 47 patients (75.8%) had at least one operation. According to the Cierny-Mader classification for anatomic type which was categorized into 11 type I, 3 type II, 37 type III and 11 type IV, 47 patients (75.8%) had good immune systems (C-M Class A hosts), while 15 patients (24.2%) had compromised locally or systemically risk factors (C-M Class B hosts). Most of the patients had no risk factor (66.1%) according to the Cierny-Mader classification such as cellulitis or abscess formation, smoking history, diabetes mellitus, anemia, chronic lung disease, poor soft tissue requiring flap, adjacent joint stiff/arthritic, or heterotopic ossification. The tibia was the most common location in 34 patients (54.8%), followed by the femur (30.6%), the humerus (6.5%), the calcaneus (3.2), the clavicle (1%), the forearm (1%) and the fibula (1%). The mean operative time was 93 min. The median number of nHA-ATB beads used was 60 beads (range 6–130 beads). The minimum number of beads used was 6 beads and the maximum number was 180 beads. The ratio of each antibiotic impregnated bead used was 1:1:1 for each case. *Staphylococcus* was the most common organism (25.8%) and followed by *Pseudomonas* spp. (12.9%) while 17 patients (54.8%) had no significant growth. The operative details are shown in Table 2.

### 2.2. Blood Biochemistry and Therapeutic Drug Monitoring

Table 3 shows blood biochemistry of patients at each follow-up period. No significant difference in CBC (data not shown), liver function or renal function results between baseline values before surgery and values at each follow-up time was seen. Although some tests might display significant differences at some follow-up, but they were clinically insignificant. However, CRP and ESR decreased with follow-up times. The values at 8 weeks were not significantly different from baseline values, but CRP and ESR reached significant differences at longer periods of 12 weeks, 24 weeks and 48 weeks. Table 4 displays the therapeutic drug monitoring of antibiotics in serum and wound drainage fluid. The vancomycin level from wound drainage fluid was 128.74 µg/mL, 175.79 µg/mL and 125.43 µg/mL at 24, 48 and 72 h after surgery, respectively. The gentamicin level from wound drainage fluid was 412.04 µg/mL, 53.03 µg/mL and 58.41 µg/mL at 24, 48 and 72 h after surgery, respectively. In contrast, the serum vancomycin level was in the range of 2.67–6.94 µg/mL while serum gentamicin level was in the range of 0.36–0.40 µg/mL which were lower than the toxic levels of 50 and 6 µg/mL for both antibiotics, respectively.

### 2.3. Treatment Outcome

**Figure 2:**
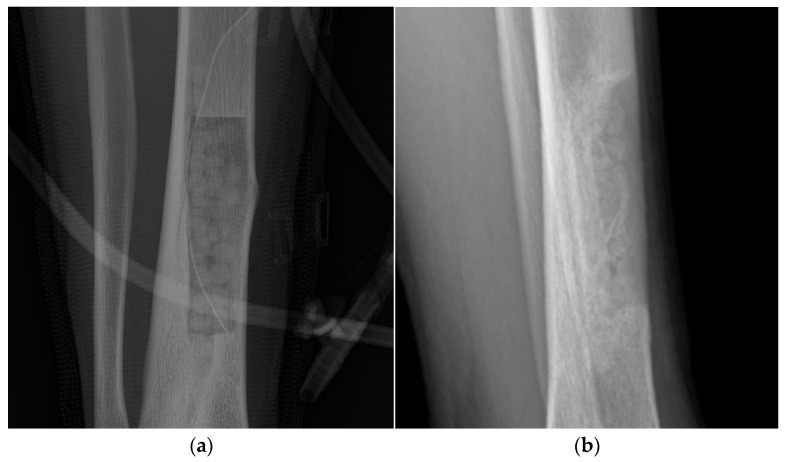
Representative radiographic images showing nHA-ATB beads in the bone cavity: (**a**) Immediate post-operatively; (**b**) 48 weeks post-operatively. The incorporation of nHA-ATB beads with surrounding bone and no translucency which might be suggestive of infection recurrence was observed.

The success rate of treatment by using nHA-ATB beads at 12 weeks was 98.28% (57/58) since one patient was reported to remain infected and the clinical outcome did not improve at 12 weeks postoperatively. The surgeon decided to discontinue this patient from the study. There was no other patient who demonstrated evidence of re-infection until 48 weeks post-operatively and the overall success rate was 98.11% (52/53). Sequential radiographs by X-ray were also performed at 6, 12, 24, and 48 weeks to determine the treatment outcome (Figure 2). Progressive improvement with follow-up times was seen in most of them without osteolytic lesions, bone destruction or sign of infection excepting one patient that was discontinued from the study. Among 53 patients, 30 patients (56.60%) were evaluated for bone union at 24 weeks after treatment. No change in alignment was seen for all of them while bone union, consolidation and callus formation were seen in twenty-one patients (70%). At 48 weeks, nHA-ATB beads were still seen in all cases without displacement or migration and there was an incorporation of the nHA-ATB beads with the surrounding bone.

### 2.4. Quality of Life by SF-36 Questionnaire

The quality of life in both physical and mental categories generally increased with follow-up periods. At 6 weeks, all domains in physical health and mental health of the patients were significantly greater than the baseline values before surgery excepting only physical function, vitality and social function which did not differ significantly from the baseline values. At 12 weeks and 24 weeks, the data demonstrated that both the physical health and mental health of patients after surgery significantly improved in all domains compared to the baseline values as shown in Table 5.

### 2.5. Adverse Events

Twenty adverse events were reported comprising 1 definitely related event, 2 possibly related events and 17 unlikely or not related events as shown in Table 6. Three related events consisted of exposure to the nHA-ATB beads from wound dehiscence, acute on-top chronic osteomyelitis and wound complication. These patients were treated with a revised wound closure and the wound later healed uneventfully. In the case of acute on-top chronic osteomyelitis event, the patient was discontinued from the study since the clinical outcome was not improved. Concerning the consequences of 20 reported adverse events, 18 events were resolved. However, two events could not be resolved including one patient who died of causes unrelated to osteomyelitis or surgery and one patient who could not be contacted to obtain the information due to loss to follow-up and was discontinued from the study.

## 3. Discussion

The success rates of osteomyelitis treatment of long bones were reported to vary between 70 and 90% with a recurrence in 6–9% of the patients depending on the severity of the injury [50,51]. Localized antibiotic delivery has been shown to have a success rate of approximately 90%, but also varied depending on several factors [16,52,53]. Generally, localized antibiotics could be used alone or in combination with antibiotics administration via intravenous (IV) injection. Gauland demonstrated that the treatment of 323 patients with confirmed chronic osteomyelitis of the lower extremity with the combination of debridement and localized delivery beads without IV antibiotics administration had the encouraging result with a success rate of 86.4% [53]. Another study demonstrated the local application of gentamicin loaded calcium sulphate/carbonate beads in which half of the patient was not administrated with antibiotics injection showed an infection control rate of 80% without nephrotoxic or hepatotoxic effects [33]. The combined treatment using both IV antibiotic administration and localized antibiotic beads was also seen to provide a better outcome than IV antibiotics administration alone. Calhoun et al. demonstrated the success rate of IV antibiotic administration was 83.3% while the combined treatment of gentamicin loaded PMMA beads, and IV drug administration yielded a success rate of 89.3% [54]. In another study, the combined treatment of gentamicin loaded PMMA beads, and IV drug administration resulted in a 100% success rate while only 95% was achieved in the antibiotics IV administration group only [55]. The use of calcium sulphate carrier containing tobramycin for the treatment of chronic osteomyelitis showed a success rate of 90.8% with the recurrent infection (9.2%) occurring at a mean of 10.3 months post-operatively. McNally et al. reported the mid- to long-term result of single-stage surgery for patients with chronic osteomyelitis using a bioabsorbable gentamicin-loaded calcium sulphate/hydroxyapatite with 94% infection control [56]. No recurrence of infection was seen after using calcium hydroxyapatite block filled with antibiotic powder in the center during follow-up ranging from 24 to 75 months was reported [45] while the success rate of antibiotic impregnated hydroxyapatite blocks ranged 85–100% at the follow-up periods of 1–6 years [46,47,48]. In this study, the success rate of treatment by using nHA-ATB at 12 weeks was 98.28% while the overall success rate at 48 weeks was 98.11%. The recurrence rate at 48 weeks was 1.89% (1/53). Although it might be argued that a high success rate in this study may be due to our patients having good physiological status (75.8% was class A host) since it was reported previously that patients with class A host showed 96% success rate, while a success rate of 74% was seen for class B host [57]. Nevertheless, it was reported in another study that the re-infection rate was not significantly related to the physiological class of the host, microbiological culture, or the presence of an infected nonunion before surgery [56]. Therefore, the infection control rate by adjuvant use of nHA-ATB beads was not inferior and was comparable with other localized systems in previous studies.

After treatment, PMMA bead chain cannot be left in place since it could prevent bone ingrowth over time and will also present a risk of secondary infection on its surface [58]. The idea of the antibiotic carrier that could remain in the wound defect without the need for subsequent removal was; thus, foreseen. Calcium sulfate was later employed as a biodegradable antibiotic carrier that showed a resorption time in the range of 3 to 12 weeks as determined radiologically [59,60,61]. However, the bone regeneration capacity following the dissolution of the calcium sulfate beads varied and remained inconclusive. It was reported that either no bone filling, partial bone filling or complete bone filling was seen, and the mean percentage of bone filling ranged 37.5–49.7% [26,62,63]. This inconsistency might be related to the resorption rate which might vary for each patient. If the rate of dissolution was fast and did not match with the osteogenesis, limited new bone formation occurred during the resorption period and led to new bone cavities [26,63]. In addition, too fast resorption rate would also release high concentrations of antibiotics at short periods to the surrounding area which might be toxic to nearby stem cells or bone cells and prevent osteogenesis [64]. Calcium phosphates, particularly hydroxyapatite is typically used as a synthetic bone substitute to regenerate a new bone in the osseous defect area. Its resorption rate was relatively lower than that of calcium sulfate and provided bone regeneration capability through its osteoconductive scaffold for cells or tissues to ingrowth and integrate. Lowering the resorption rate of calcium sulfate by mixing with calcium phosphates to allow sufficient time for the bone to grow was found to show a significantly higher percentage of new bone formation than calcium sulfate alone at 1 and 6 months [63]. In this study, nHA-ATB beads were still radiologically seen at 48 weeks postoperatively without obvious evidence of resorption. However, follow-up radiographs showed that there was the incorporation of the nHA-ATB with surrounding bones as could be seen from the loss of margins definition and internal architecture of the beads [65]. This agrees with the previous study there was progress in the incorporation of bone and antibiotic-filled sintered hydroxyapatite blocks with times for up to five years without radiological evidence of resorption and some sclerotic areas were developed around the blocks [45]. In addition, some histological studies in those re-operated patients after healing revealed some degradation of the sintered hydroxyapatite blocks through a cell-mediated process.

Inflammation can occur as either an acute phase resulting from injury or infection or a chronic phase. Blood tests including ESR and CRP are simple, rapid, and economical means that are helpful in diagnosing and monitoring inflammation. Elevated ESR was found to be associated with chronic pain patients. CRP is a protein that emerged in plasma during infectious or inflammatory conditions and participated in the acute or first phase of the inflammatory process. In addition, these tests can be used as indicators for the presence of pain and inflammation and markers of treatment effectiveness. The osteomyelitis diagnosis by using ESR and CRP as biomarkers were also reported by many studies, but the correlation success and cut-off values varied depending on the locations and risk factors [66,67,68,69]. Generally, the combined use of both ESR and CRP was recommended to gain sufficient specificity and it was regarded as an adjunct tool for diagnosis or monitoring the treatment success rather than the definite diagnosis [70,71]. In this study, both CRP and ESR of chronic osteomyelitis patients were observed to continuously and significantly decreased during follow-up periods after surgical treatment. This corresponded well with the clinical and radiological evaluation that the number of patients who recovered from osteomyelitis increased from 4 at 8 weeks to 31 at 48 weeks. The overall number of patients whose conditions were either recovered or improved ranged from 51–58 patients at 48 weeks follow-up which accounted for 83.6–95.08% indicating the efficacy of the treatment. Only one patient was diagnosed as worsening but was not indicated as directly related to nHA-ATB beads used and might be caused by other complications or causes.

Psychosocial complications resulting from chronic osteomyelitis are common and it was mainly caused by loss of working ability, loss of medical coverage and loss of family support which in turn was significantly affected by the number of surgical procedures or unsuccessful antibiotic treatments [72]. It was observed that the quality of life of vertebral osteomyelitis patients improved significantly at 1 year post-operatively, but non-significantly changed from year 1 to year 2 [73]. In another study, the quality of life of patients in the treatment success group did not differ significantly from the treatment failure group [74]. In this study, the quality of life of patients after treatment improved significantly compared to the baseline before surgery in all domains including both physical health and mental health after 12 weeks and 24 weeks. However, some domains of physical function, vitality, and social function did not differ significantly at 6 weeks which was possibly caused by the intervention, which might limit the movement and activity of the patient in the early stage after surgery.

The pharmacokinetics of the treatment in this study were monitored by measuring the levels of vancomycin and gentamicin in serum and wound drainage fluid at 24 h, 48 h and 72 h after surgery. Fosfomycin was not determined in this study since no toxicity level was established. A high concentration of vancomycin and gentamicin in wound drainage fluid was detected at all periods whereas limited antibiotics concentration was found in serum even as high as 180 beads were used in one case and all the antibiotics levels were lower than the toxicity level of vancomycin and gentamicin which were 50 µg/mL and 6 µg/mL respectively. This demonstrated that high concentrations of antibiotics were locally released at the bone defect as intended, without systemic release into the bloodstream, and exposing the patient to antibiotic side effects. Correspondingly, no side effect of antibiotics was reported in all patients during the study. This was in accordance with previous studies that high concentrations of gentamicin which was 10–100 times greater than the MIC of bacteria were detected in the wound drainage fluid of the patients when being treated by gentamicin loaded polymethylmethacrylate beads [75,76]. In contrast, the low serum gentamicin level which was not greater than 0.5 µg/mL was found even as high as 80–180 gentamicin loaded polymethylmethacrylate beads were placed [75,77]. High tissue levels and low serum levels of antibiotics in patients after treatment were substantiated for calcium sulfate beads as well [78,79]. The pharmacokinetics of the treatment by using nHA-ATB beads correlated well with the blood chemistry test especially liver function and renal function which showed no significant difference at all follow-up indicating no nephrotoxic or hepatotoxic effects were found after infection treatment by using nHA-ATB beads.

Concerning the reports of complications and adverse events in relation to the use of local antibiotic beads. In the case of antibiotic loaded PMMA bead chain, mechanical complications including damage to the bowel or veins, inability to reduce a hip dislocation secondary and bead migration were reported [80,81,82]. There have also been reports of increased antibiotic resistance when PMMA bead chains were implanted for an extended period [21]. Regarding the calcium sulfate beads, three main complications which were related to the beads included prolonged wound drainage, transient hypercalcemia, and heterotopic ossification. The incidences rate of these complications was 3.2–51%, 5.4–20% and 1.7% respectively. These complications were hypothesized to occur due to the fast resorption of calcium sulfate beads which led to a calcium-rich fluid in the wound area, and it was found to directly correlate with the volume of implanted calcium sulfate beads especially for wound drainage and hypercalcemia [24,26,59,61,83,84,85,86]. It was even suggested that precautions against hypercalcemia which may cause convulsions, coma and cardiac arrest should be implemented and regulate the number of beads used (40 cc per intervention or 80 cc if used intramedullary) [24,87]. Other complications that could be found included experiencing pain and discomfort [88]. In this study, twenty adverse events were encountered for nHA-ATB. Among these, only one event (5%, 1/20) was reported to be definitely related to nHA-ATB beads, which was the exposure of beads, but the severity was low. Two possibly related events (10%, 2/20) including serum oozing from the surgical wound and acute on top chronic left tibia osteomyelitis were also observed. The overall incidence rate was thus 4.84%. Most of the events were resolved without having to remove the nHA-ATB beads and the patients recovered. No other complications as seen in the case of using PMMA or calcium sulfate were encountered. These all supported the safety of using nHA-ATB beads compared to other commercial products.

The advantages of using nHA-ATB beads as a local antibiotics carrier were; thus, their ability to provide a sustained, local, high concentration of antibiotics at the site of infection and lower systemic side effects compared with intravenous antibiotics. It could be left inside the body without the need for subsequent removal and act as a bone graft to provide bone regeneration capability in the bone defect. However, the drawbacks of using local antibiotics beads such as the nHA-ATB beads in this study compared to the standard intravenous antibiotics treatment would be the additional cost of the product and additional surgical time in filling the defects with the beads.

Limitations of this study were that the follow-up time is relatively short and no direct comparison with standard treatment or analysis of the risk of treatment failure was implemented. Osteomyelitis may recur years later, despite what appears to be a successful recovery. A longer follow-up period is needed to avoid overestimating the efficacy of the treatment. A future randomized controlled trial is considered to improve understanding of the efficacy and safety outcomes of using nHA-ATB beads in chronic osteomyelitis patients. In addition, the cost of the treatment analysis by using nHA-ATB beads in comparison to standard treatment should be performed to justify the financial benefit of using it as an alternative product of choice for chronic osteomyelitis treatment.

## 4. Materials and Methods

### 4.1. Patient Enrollment

This study was performed in accordance with the Declaration of Helsinki and the International Conference on Harmonization (ICH) for Good Clinical Practice (GCP). The protocol was registered in the Thai Clinical Trials Registry (TCTR20170123002) and approved by the human research ethical committee in each participated center. Patients with chronic osteomyelitis in 12 participating centers in Thailand including Thammasat university, Bhudasothon hospital, Lerdsin hospital, Srinakharinwirot university, Lampang hospital, Khon Kaen hospital, Pathumtani hospital, Suppasitthiprasong hospital, Bhumibol Adulyadej hospital, Paholpolpayuhasena hospital, Hatyai hospital and Prince of Songkla university were eligible to enroll in this multicenter, prospective cohort study. The sample size was calculated using a confidence interval for a proportion (http://www.sample-size.net/sample-size-conf-interval-proportion/, accessed on 16 March 2020) and 59 patients were necessary to provide a study power of 90% with an α of 0.10. Assuming a 5% loss to follow-up for 48 weeks period; hence, the number of participants was 62 patients.

The inclusion criteria were the age equal or above 18 years old and diagnosed as chronic osteomyelitis. A sinus, an abscess, intraoperative pus, supporting histology, or two or more microbiological cultures with identical organisms were all required for the diagnosis of chronic osteomyelitis, which was defined as the existence of symptoms for at least six weeks [89,90]. The exclusion criteria were the patient with a history of vancomycin, gentamicin or fosfomycin allergy, renal insufficiency, immunocompromise host, multiple active injuries or head injuries, malignancy at the site of infection, poor control of diabetes (HbA1C > 8 mg%), pregnancy, human immunodeficiency virus (HIV) infection, infection with antibiotic-resistant gram-negative bacteria. The participant will be discontinued from the study if they lose follow-up 2 times consecutively, the drug level is above the toxicity level (vancomycin > 50 µg/mL, gentamicin > 6 µg/mL), presence of drug allergy symptoms during the study and antibiotic-resistant gram-negative bacteria from intra-operative culture. The demographic data including sex, age, wound location, Cierny-Mader (C-M) (anatomic type, physiological condition and risk factors) [89], causes and history of previous surgery for osteomyelitis were collected.

### 4.2. Preparation of Antibiotic Impregnated Microporous Nanohydroxyapatite Beads

Antibiotic impregnated microporous nanohydroxyapatite (nHA-ATB) beads were prepared as described in the previous study [43]. Premixed calcium sulfate-based powder was loaded into a powder-based three-dimensional (3D) printing machine (Z400, Z Corporation, Burlington, MA, USA) to print spherical beads having a diameter of 7 mm by using water-based binder (Zb 7, Z Corporation, USA) as a jetting media and a layer thickness of 0.1 mm. The as-fabricated calcium sulfate beads were then transformed to hydroxyapatite by immersing them in 1 M of disodium hydrogen phosphate solution at 80 °C for 48 h. After reaching the period, the beads were taken out, cleaned by sonication in deionized water and oven dried. The compressive strength of nHA was 0.60 MPa and its porosity and mean pore size were 63.92% and 0.15 microns, respectively [43]. nHA beads were impregnated with one of three types of antibiotics intravenous injection solution including gentamicin, vancomycin or fosfomycin using a vacuum-assisted method under the same condition each time. After impregnation, the samples were taken out and dried in the room atmosphere for 48 h. The representative samples were randomly selected to measure antibiotic content in the beads to make sure that the preparation is consistent. They were then packed in a Tyvek heat-seal sterilization pouch, sterilized by ethylene oxide gas, and kept in dry conditions at room temperature until use.

### 4.3. Operative Procedure

**Figure 3:**
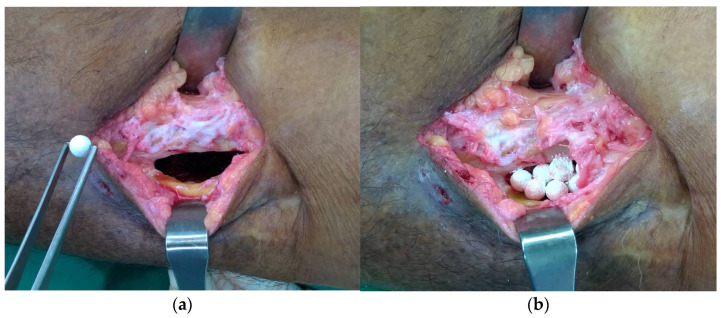
Intra-operative photographs showing the debridement and placement of nHA-ATB into the bone cavity: (**a**) The infected bone was curetted and was irrigated with copious normal saline; (**b**) The bone cavity was completely filled with nHA-ATB beads.

The patient was under general anesthesia or regional anesthesia depending on the location of the infected bone. The sterile preparation was done, and the sinus tract was excised. The debridement was down to the infected bone. Any dead bone and non-vitalized tissue were removed. The multiple deep tissue samplings were collected for microbiological study before the administration of intravenous antibiotics. The cultured isolates were identified by colony characteristics and biochemical reactions. Antibiotic sensitivity was done by Kirby Bauer disc diffusion method on Mueller Hinton agar as per Clinical Laboratory Standards Institutes (CLSI) guidelines. The infected bone was curetted with a mechanical burr until the healthy bleeding bone was reached and then was copiously irrigated with normal saline until no gross infection was present. The nHA-ATB beads were placed into the defect site at an equal ratio (1:1:1) of each antibiotic to completely fill the defect (Figure 3). The rationale of using three antibiotics in this study was to deliver broad-spectrum antibiotics locally to cover the most common organisms generally found in chronic osteomyelitis. Gel foam was applied to cover the beads securely within the intramedullary canal or defect. Hemostasis was achieved and the radivac drain was inserted. The subcutaneous and skin were closed, respectively. Flap was considered in case of inadequate soft tissue coverage. The intravenous cephalosporin was administered immediately after tissue sampling and the appropriate antibiotic was adjusted as soon as the culture result was obtained for 1 to 6 weeks or longer as advised by the infectious disease team. Therapeutic drug monitoring of gentamicin and vancomycin was performed on thirty-four patients (30% of the total number of patients) from Thammasat university or the Prince of Songkla university. Blood samples (10–15 mL) and wound drainage fluid via a radivac catheter were collected at 24 h, 48 h, and 72 h after surgery.

The follow-up evaluations were scheduled at 2 weeks, 4 weeks, 6 weeks, 12 weeks, 24 weeks, and 48 weeks postoperatively. The laboratory test including CBC, ESR, CRP, liver function test and renal function was done at 6 weeks, 12 weeks, 24 weeks, and 48 weeks. The radiographic examination of the treated site was performed to evaluate the radiographic union and the resorption of the materials. Adverse events and complications were recorded at all time points. All patients gave informed consent.

The primary endpoints of the study were the success rate at 12 weeks and the re-infection rate within 24 weeks period after the operation. The success of treatment was defined as no recurrent infection, no recurrence of sinus drainage, normal level of ESR and CRP or no requirement of antibiotics for persistent symptoms during the follow-up. The secondary endpoints were the quality of life of the participants at 0, 6, 12 and 24 weeks postoperatively which was evaluated by the Thai version of the SF-36 questionnaire [91] and the incidence of adverse effects which was graded according to National Cancer Institute criteria [92].

### 4.4. Statistical Analysis

All the data were analyzed using statistical analysis software (STATA version 14, StataCorp LLC, College Station, TX, USA). The data were described as the mean ± standard deviation. Blood biochemistry and quality of life were compared before and after surgery for each participant using a paired t-test. A *p*-value < 0.1 was considered statistically significant.

## 5. Conclusions

Microporous nanohydroxyapatite beads impregnated with vancomycin or gentamicin or fosfomycin could be employed as an alternative product of choice for chronic osteomyelitis treatment. Within the scope of this study, good outcomes in infection treatment were observed throughout the follow-up period and the material was safe and well tolerated while providing the enhancement in quality of life of the patients.

## Figures and Tables

**Table 1:** Demographic data.

Characteristics *n* = 62	*n* (%)
Sex	
Male	49 (79.0)
Female	13 (21.0)
Age (Mean ± SD)	47.2 ± 14.7
Location	
Tibia	34 (54.8)
Femur	19 (30.6)
Humerus	4 (6.5)
Calcaneus	2 (3.2)
Clavicle	1 (1.6)
Forearm	1 (1.6)
Fibula	1 (1.6)
Cierny-Mader Grade	
Type I Medullary osteomyelitis	11 (17.7)
Type II Superficial osteomyelitis	3 (4.8)
Type III Localized osteomyelitis	37 (59.7)
Type IV Diffuse osteomyelitis	11 (17.7)
Physiological class	
Class A Good immune system and delivery	47 (75.8)
Class B Compromised locally or systemically risk factors	15 (24.2)
Class C requires suppressive or no treatment; minimal disability	0 (0)
Causes	
Post-traumatic	48 (77.4)
Hematogones (non-trauma, sepsis)	14 (22.6)
Previous surgery for this condition	
0	15 (24.2)
1	19 (30.6)
2	13 (21.0)
3	8 (12.9)
4	2 (3.2)
5	2 (3.2)
6	1 (1.6)
>7	2 (3.2)
Risk factor	
No risk factor	41 (66.1)
Risk	21 (33.9)
Major risk factor: cellulitis or abscess formation	5 (23.8)
Minor systemic risk factor: subject had smoking history	7 (33.3)
Minor systemic risk factor: diabetes mellitus	3 (14.3)
Minor systemic risk factor: anemia	2 (9.5)
Minor systemic risk factor: chronic lung disease	1 (4.8)
Minor local risk factor: poor soft tissue requiring flap	9 (42.9)
Minor local risk factor: adjacent joint stiff/arthritic	7 (33.3)
Minor local risk factor: heterotopic ossification	1 (4.8)

**Table 2:** Surgical details and cultured results.

Surgery Data	Quantity (Median, IQR (Min-Max))
d	93, 57
Number of antibiotics loaded hydroxyapatite beads used	60, 54 (6–180)
Gentamicin beads	20, 18 (2–60)
Vancomycin beads	20, 20 (2–60)
Fosfomycin beads	20, 20 (2–60)
Cultured organism	*n* (%)
No	3 (4.8)
Yes	59 (95.2)
No growth	28 (47.5)
Cultured organism (2 organisms found)	7 (11.9)
*Acinetobactor baumannii* and *E. coli* (ESBL-Producing)	1 (14.3)
*Acinetobactor baumannii* and *Pseudomonas aeruginosa*	1 (14.3)
*Morganella morganii* and *Klebsiella pneumoniae* (MDR)	1 (14.3)
*Pseudomonas aeruginosa* (MDR) and *Staphylococcus haemolyticus* (MR-CoNS)	1 (14.3)
*Pseudomonas aeruginosa* and *Enterococcus avium*	1 (14.3)
*Pseudomonas aeruginosa* and *Shewanella putrefaciens*	1 (14.3)
*Staphylococcus aureus* and *Staphylococcus epidermidis*	1 (14.3)
Cultured organism (1 organism found)	24 (40.7)
*Pseudomonas aeruginosa*	9 (37.5)
*Staphylococcus aureus*	4 (16.7)
*Staphylococcus aureus* (MRSA)	1 (4.2)
Aerobic culture	1 (4.2)
*Aerococcus viridans*	1 (4.2)
*Coagulase Negative Staphylococci*	2 (8.4)
*Enterobacter cloacae*	1 (4.2)
*Enterococcus faecalis*	1 (4.2)
*Serratia marcescens*	1 (4.2)
*Staphylococcus cohnii*	1 (4.2)
*Staphylococcus haemolyticus*	1 (4.2)
*Staphylococcus hominis*	1 (4.2)
Vancomycin sensitivity (*n* = 38)	
Sensitive	8 (21.1)
Intermediate	0 (0)
Resistant	0 (0)
Results not reported by hospital lab	30 (78.9)
Gentamicin sensitivity (*n* = 38)	
Sensitive	19 (50)
Intermediate	1 (2.6)
Resistant	4 (10.5)
Results not reported by hospital lab	14 (36.8)
Fosfomycin sensitivity (*n* = 38)	
Sensitive	9 (23.7)
Intermediate	1 (2.6)
Resistant	2 (5.3)
Results not reported by hospital lab	26 (68.4)

IQR = Interquartile range.

**Table 3:** Blood biochemistry.

Results	Baseline Mean ± SD	8 Weeks Mean ± SD	12 Weeks Mean ± SD	24 Weeks Mean ± SD	48 Weeks Mean ± SD
Liver function test					
AST (SGOT) (U/L)	29.31 ± 13.85	30.25 ± 16.02	27.53 ± 12.04	31.08 ± 14.85	29.37 ± 14.75
ALT (SGPT) (U/L)	32.78 ± 29.95	34.5 ± 29.32	28.81 ± 24.86	31.67 ± 22.80	27.07 ± 19.47
Alkaline Phosphatase (ALP) (U/L)	103.36 ± 47.29	103.81 ± 34.84	97.96 ± 32.60	94.37 ± 33.25	83.44 ± 25.98
Renal function test					
Serum Creatinine (mg/dL)	0.81 ± 0.20	0.85 ± 0.25	1.13 ± 1.74	0.85 ± 0.21	0.88 ± 0.20 ^#^
eGFR (ml/min/1.73 m^2^)	103.11 ± 21.84	97.67 ± 25.52 ^#^	98.55 ± 23.59 ^#^	100.46 ± 19.9	96.58 ± 20.86 ^#^
BUN (mg/dL)	14.03 ± 13.28	12.24 ± 4.47	11.65 ± 3.64	13.22 ± 4.28	12.90 ± 3.80
C-reactive protein (CRP) (mg/dL)	12.30 ± 27.82	3.96 ± 4.86	3.12 ± 4.52 ^#^	2.7 ± 4.11 ^#^	2.45 ± 2.65 ^#^
Erythrocyte sedimentation rate (ESR) (mm/hour)	45.11 ± 32.83	40.09 ± 26.53	27.37 ± 21.68 ^#^	26.72 ± 27.67 ^#^	25.13 ± 26.70 ^#^

^#^ Denoted significantly different compared to the baseline values, *p* < 0.1.

**Table 4:** Therapeutic drug monitoring of antibiotics level.

	Antibiotics	Duration (h)	Concentration (µg/mL) Mean ± SD
Wound drainage fluid	Vancomycin	24	128.64 ± 134.49
		48	175.79 ± 153.96
		72	125.43 ± 159.87
	Gentamicin	24	412.04 ± 646.01
		48	53.03 ± 74.06
		72	58.41 ± 97.81
Serum	Vancomycin	24	4.00 ± 0.00
		48	2.67 ± 2.31
		72	6.94 ± 4.16
	Gentamicin	24	0.38 ± 0.17
		48	0.36 ± 0.12
		72	0.40 ± 0.00

**Table 5:** Quality of life of participants at each follow-up as evaluated by SF-36 questionnaire.

Results	Baseline Mean ± SD	6 Weeks Mean ± SD	12 Weeks Mean ± SD	24 Weeks Mean ± SD
Physical health	37.14 ± 4.23	47.94 ± 14.77 ^#^	55.51 ± 13.78 ^#^	60.63 ± 14.64 ^#^
Physical functioning	46.86 ± 26.10	50.49 ± 26.91	63.98 ± 26.12 ^#^	72.93 ± 23.49 ^#^
Role-physical	5.27 ± 8.14	11.52 ± 10.71 ^#^	14.29 ± 10.67 ^#^	16.98 ± 9.65 ^#^
Bodily pain	46.96 ± 26.56	64.39 ± 20.34 ^#^	73.47 ± 18.29 ^#^	78.89 ± 19.95 ^#^
General health	49.45 ± 19.50	65.35 ± 17.69 ^#^	70.31 ± 16.84 ^#^	73.72 ± 17.99 ^#^
Mental health	47.44 ± 17.48	58.99 ± 14.12 ^#^	64.81 ± 12.83 ^#^	67.21 ± 13.63 ^#^
Vitality	66.79 ± 24.48	80.76 ± 21.24	87.88 ± 19.53 ^#^	91.44 ± 20.89 ^#^
Social function	56.62 ± 25.54	67.40 ± 20.78	75.00 ± 19.09 ^#^	80.16 ± 19.82 ^#^
Role- emotional	7.35 ± 9.67	15.03 ± 10.41 ^#^	17.01 ± 10.06 ^#^	18.12 ± 10.14 ^#^
Mental health	58.98 ± 21.99	72.78 ± 14.24 ^#^	79.35 ± 12.09 ^#^	79.13 ± 13.38 ^#^

^#^ Denoted significantly different compared to the baseline values, *p* < 0.1.

**Table 6:** Adverse events and their relations to nHA-ATB beads.

Relations to nHA-ATB Beads	Adverse Events (*n* = 20)		
	SAE or Non-SAE	Severity	Events
Definitely related			
- HA exposed from wound dehiscence	Non-SAE	Grade I	1
Possibly related			
- Serum oozing from the surgical wound	Non- SAE	Grade I	1
- Acute on-top chronic left tibia osteomyelitis	SAE	Grade III	1
Unlikely			
- Anterior shoulder dislocation	Non-SAE	Grade II	1
- Loosening of external fixator	SAE	Grade I	1
- Gastrointestinal hemorrhage, unspecified	SAE	Grade II	1
Not related			
- Leg pain	Non-SAE	Grade I	1
- Wrist drop due to radial nerve palsy	Non-SAE	Grade II	1
- Acute pyelonephritis	SAE	Grade I	1
- Adjust external fixation	SAE	Grade I	1
- Bone fracture	SAE	Grade I	1
- Prolonged hospitalization of chronic osteomyelitis of left distal femur due to financial problem	SAE	Grade I	1
- Revised external fixation	SAE	Grade I	1
- Wound dehiscence	SAE	Grade I	2
- Surgical wound with drainage	SAE	Grade II	1
- Wound dehiscence after debridement and switch HA-ATB to cement ATB	SAE	Grade II	1
- Broken Steinman Pin	SAE	Grade III	1
- Nonunion of the fracture site	SAE	Grade IV	1
- Death from other causes	SAE	Grade V	1

## Data Availability

The data presented in this study are available on request from the corresponding author. The data are not publicly available due to ethical restrictions.
